# Positive Relationship Between Paroxysmal Vertigo and Right-to-Left Shunt: A Large Observational Study

**DOI:** 10.3389/fneur.2022.927853

**Published:** 2022-06-03

**Authors:** Kaiming Liu, Xiulin Tian, Wenwu Hong, Yujin Xiao, Juanyan Chen, Haidi Jin, Faming Wang, Xiaopei Xu, Tao Zang, Liang Zhang, Mengxiong Pan, Xiaodong Zou

**Affiliations:** ^1^Department of Neurology, School of Medicine, Second Affiliated Hospital, Zhejiang University, Hangzhou, China; ^2^Department of Neurology, Tiantai People's Hospital of Zhejiang Province, Taizhou, China; ^3^Department of Neurology, Jiaxing Hospital of Traditional Chinese Medicine, Jiaxing, China; ^4^Department of Neurology, Dongyang People's Hospital, Dongyang, China; ^5^Department of Neurology, Wanna Medical College, Wuhu, China; ^6^Department of Neurology, Tongxiang Second People's Hospital, Tongxiang, China; ^7^Department of Neurology, First People's Hospital of Huzhou, Huzhou, China; ^8^Department of Neurology, Tongde Hospital of Zhejiang Province, Hangzhou, China

**Keywords:** benign recurrent vertigo, contrast transthoracic echocardiography, paroxysmal vertigo, right-to-left shunt, vestibular migraine

## Abstract

**Background:**

The association between paroxysmal vertigo and right-to-left shunt (RLS) is rarely reported. This study investigates the prevalence and correlation of RLS in patients with different paroxysmal vertigo diseases.

**Methods:**

Patients with paroxysmal vertigo from seven hospitals in China were included in this observational study between 2017 and 2021. Migraine patients within the same period were included for comparison. Demographic data and medical history were collected; contrast transthoracic echocardiography was performed; and the clinical features, Dizziness Handicap Inventory, and incidence of RLS in each group were recorded.

**Results:**

A total of 2,751 patients were enrolled. This study's results demonstrated that the proportion of RLS in patients with benign recurrent vertigo (BRV) and vestibular migraine (VM) was significantly higher than that in patients with benign paroxysmal positional vertigo, Meniere's disease, and vestibular paroxysmia (*P* < 0.05). No statistical difference was shown between the frequency of RLS in patients with BRV and those with migraine and VM. A positive correlation was shown between the RLS grade and Dizziness Handicap Inventory scores of patients with VM and BRV (*P* < 0.01) after effectively controlleding the effect of confounding variables.

**Conclusions:**

RLS was significantly associated with BRV and VM. RLS may be involved in the pathogeneses of BRV and VM and may serve as a differential reference index for the paroxysmal vertigo.

**Trial Registration:**

CHRS, NCT04939922, registered 14 June 2021- retrospectively registered, https://register.clinicaltrials.gov.

## Introduction

Paroxysmal vertigo resulting from different diseases has distinct clinical symptoms and signs. However, significant overlaps exist in the clinical symptoms of different vestibular diseases, such as vestibular migraine (VM), which may have manifestations similar to Meniere's disease or benign paroxysmal positional vertigo (BPPV) (1). Furthermore, some patients with VM may have no headache, while Meniere's disease may be accompanied by migraine, photophobia, phonophobia, and other symptoms. This increases the difficulty in distinguishing these diseases from one another (2, 3).

Benign recurrent vertigo (BRV) was first described by Slater as a group of clinical syndromes with recurrent vertigo without nervous system and cochlear symptoms (4). Currently, no recognized diagnostic criteria for paroxysmal vertigo exist; thus, diagnosis is often made by exclusion (5). Follow-up studies have shown that BRV is closely associated with migraine, as 51–87% of BRV cases are comorbid with migraine (6, 7). This proportion of BRV meets the description of a definite or possible VM.

BRV and VM differ in terms of their definition and diagnostic criteria. BRV can have no headache and migraine, while VM requires at least a history of migraine or migraine-like symptoms (8). Given that BRV and VM have no specific physical or auxiliary examination findings, clinical diagnosis is often challenging. Therefore, identifying anatomic features related to certain forms of paroxysmal vertigo that may aid in establishing a diagnosis could help clinicians make a correct diagnosis. Such identification could also help determine the pathogenesis of diseases with similar clinical symptoms.

Previous studies have found that patent foramen ovale (PFO) prevalence in patients with migraine is significantly higher than that in healthy individuals (9, 10). The relationship between PFO and migraine has been studied and is a major concern (11). However, there is no good quality evidence to support a link between migraine and PFO. VM is related to vestibular symptoms caused by migraine mechanisms, and its pathogenesis may be related to ion channel defects, enhanced cortical excitability, and central sensitization, which are caused by genetic susceptibility (12, 13).

PFO is the most common cause of right-to-left shunt (RLS), which refers to the potentially abnormal channels between the left and right atrium and ventricle or systemic and pulmonary circulations. RLS can be categorized as intracardiac shunt or extracardiac based on its location. Intracardiac shunt includes PFO, while extracardiac includes pulmonary arteriovenous malformation and patent ductus arteriosus.

To date, there is little published research on the relationship between RLS and paroxysmal vertigo. Diagnostic tests used for detecting RLS include contrast transcranial Doppler, contrast transthoracic echocardiography (cTTE), and contrast transoesophageal echocardiography. Given that cTTE is easy to perform and has high sensitivity and specificity (14), we chose this method to investigate the prevalence of paroxysmal vertigo (VM with headache, VM without headache, BRV, Meniere's disease, BPPV, and vestibular paroxysmia (VP)) in adults with RLS compared to patients with migraine. This helped evaluate the correlation between RLS and different types of paroxysmal vertigo diseases.

## Methods

### Study Design and Patient Population

**Figure 1:**
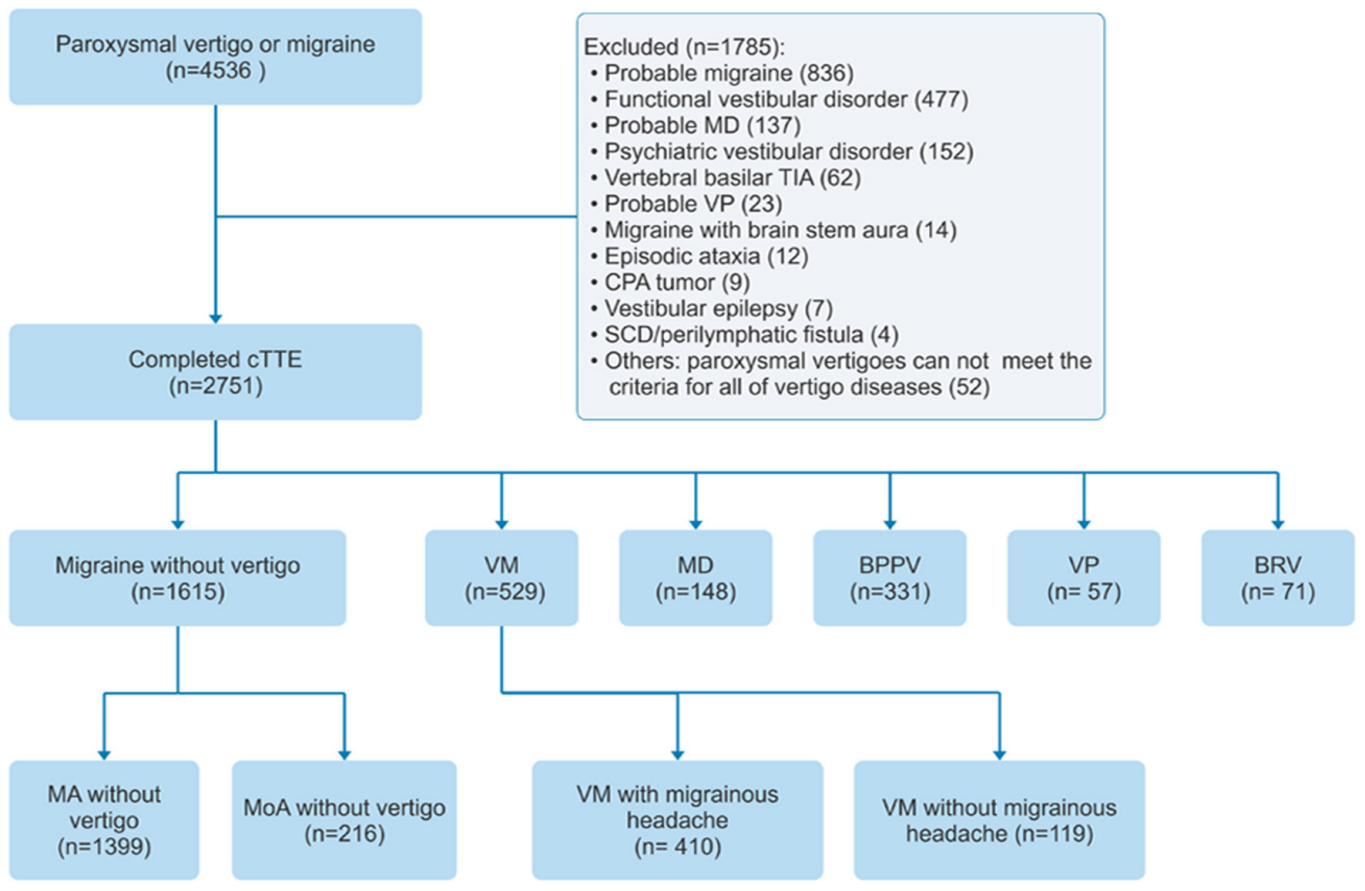
Flowchart of the inclusion and exclusion of patients.

Patients from the headache and vertigo clinics of seven hospitals in China were included in the study between July 2017 and July 2021 (ClinicalTrial.gov ID: NCT04939922). The study used a consecutive sampling of patients (*n* = 4,536) with migraine or paroxysmal vertigo aged >18 years. Patients were screened for VM, pure BRV, definite Meniere's disease, BPPV, VP, migraine with aura, and migraine without aura and, if positive, were included in the study. Patients who were diagnosed with probable migraine, psychiatric vestibular disorder, probable Meniere's disease, probable VP, functional vestibular disorder, vertebral basilar transient ischemic attack, migraine with brainstem aura, episodic ataxia, cerebellopontine angle tumor, vestibular epilepsy, or superior canal dehiscence/perilymphatic fistula, and patients with other paroxysmal vertigo who did not meet the diagnostic criteria of all other vertigo diseases, were excluded. A flowchart of patient inclusion and exclusion is depicted in Figure 1.

The inclusion criteria for patients with VM met the definite and probable diagnostic criteria for VM established by the International Bárány Society and the International Headache Society (8). The inclusion criteria for BRV in this study were based on the exclusive diagnostic method proposed in the literature (15) and a personal history of no migraine headache, visual aura, photophobia, or phonophobia (to distinguish and exclude definite VM and probable VM), which we defined as pure BRV here (Table 1).

**Table 1:** Inclusion criteria for pure benign recurrent vertigo.

More than two attacks of spontaneous rotational vertigo that does not occur during the head movements or positional changes
No associated migrainous headaches or auditory symptoms during or between the attacks No associated photophobia and phonophobia No associated visual aura
No associated focal neurologic symptom during the attack or afterward suggesting episodic ataxia, transient ischemic attack, or vestibular epilepsy
No evidence of peripheral vestibulopathy on head-impulse, caloric, and rotatory tests
No asymmetric hearing impairment documented in pure-tone audiometry
No lesions on brain magnetic resonance imaging responsible for the benign recurrent vertigo
No history of disorders that may explain the recurrent vertigo
Not better accounted for by another vestibular disorder, including compensated vestibular neuritis, vestibular migraine, Meniere's disease, benign paroxysmal positional vertigo, and vestibular paroxysmia

The selection criteria for patients with definite Meniere's disease, BPPV, and VP were in accordance with the diagnostic criteria established by the Bárány Association, and the selection criteria for migraine were consistent with the diagnostic criteria of the International Classification of Headache Disorders, 3^rd^ edition (16–19). As RLS is strongly related to migraine with auras, migraine was further divided into migraine with aura and migraine without aura. VM was further divided into VM with and without migrainous headache. All patients with VP selected in this study were responsive to carbamazepine treatment. All patients were independently diagnosed by two experienced neurologists to reduce bias.

The patients included in this study underwent cTTE and were assigned to one of eight groups based on diagnosis: VM with migrainous headache, VM without migrainous headache, pure BRV, definite Meniere's disease, BPPV, VP, migraine with aura, and migraine without aura. This study was approved by the ethics committees of the participating centers, and all patients provided written informed consent.

### Contrast Transthoracic Echocardiography Inspection Method

While the patients were in the supine position, venous access was established by conventional elbow venipuncture, and a three-way tube was connected to the puncture site. The contrast medium was formulated after 8 mL saline, 1 mL venous blood, and 1 mL air were fully injected and concussed through the tee tube. The contrast was quickly injected while the patient was at rest and the Valsalva maneuver was performed. The apical four-chamber section was selected, and the number of microbubble signals in the left cardiac system was observed within 10 cardiac cycles after the right cardiac system was filled with microbubble signals. RLS of the heart was considered when there was more than one microbubble in the left cardiac cavity.

The RLS was graded quantitatively according to the number of microbubbles in the left ventricular cavity after the Valsalva maneuver. The grading standard is as follows: grade 0: no microbubbles in the left cardiac cavity and no RLS; grade I: 1–10 microbubbles/frame were seen in the left ventricular cavity and a small amount of RLS; grade II: 10–30 microbubbles/frame were seen in the left cardiac cavity and a medium amount of RLS; and grade III: more than 30 microbubbles/frame was seen in the left ventricular cavity, or the left ventricular cavity was almost full of microbubbles (20).

RLS from PFO was considered when microbubbles were found within 3–5 cardiac cycles, while RLS from pulmonary arteriovenous malformations was considered when microbubbles were found in more than five cardiac cycles during the cTTE examination (21).

### Dizziness Handicap Inventory

The quality of life measure for vestibular disorders was assessed using the 25-item Dizziness Handicap Inventory (DHI), which has three response categories: the functional, emotional, and physical aspects of life (22). The total score ranges from 0 (not handicapped) to 100 (severely handicapped).

### Statistical Analysis

Data measurements assumed a normal distribution and are expressed as x ± s. We used the χ^2^ test and *t*-test (or Mann–Whitney *U*-test) to compare categorical and continuous variables. Spearman's correlation coefficient was used to analyse the correlation between the DHI and RLS grades. The level of statistical significance was set at *P* < 0.05. All analyses were performed using SPSS software version 20 (IBM Corp., Armonk, NY, USA).

## Results

### Patient Demographic and Clinical Characteristics at Baseline

Within the time frame, a total of 2,751 patients completed cTTE at the outpatient departments of the seven participating centers. The mean age was 43.16 ± 14.58 years, and 65.18% were women. The demographics and clinical characteristics of the patients are summarized in Table 2.

**Table 2:** Demographic and clinical characteristics of patients at baseline.

	**MoA without vertigo** **(*n* = 1,399)**	**MA without vertigo** **(*n* = 216)**	**VM with** **migrainous headache** **(*n* = 410)**	**VM without** **migrainous headache** **(*n* = 119)**	**MD** **(*n* = 148)**	**BPPV** **(*n* = 331)**	**VP** **(*n* = 57)**	**BRV** **(*n* = 71)**
Sex (female, %)	73.27	75.46	76.83	72.27	57.43	64.65	59.65	71.83
Age (mean±SD)	39.85 ± 15.47	37.7 ± 15.41	42.67 ± 12.56	36.67 ± 14.99	52.35 ± 7.28	56.13 ± 10.48	53.74 ± 6.15	50.7 ± 18.93
Duration of attacks								
<1			45	17	0	331	47	7
<5	NA	NA	94	15	0	0	10	13
<1			128	29	85	0	0	26
<24			118	41	63	0	0	18
<24			25	17	0	0	0	7
Migrainous headache	1,399	216	410	0	0	0	0	0
Aura	0	216	53	16	0	0	0	0
Photophobia	992	156	386	107	0	2	1	3
Phonophobia	984	167	389	110	1	0	0	1
Photophobia and phonophobia	961	144	372	101	0	0	0	0

*BPPV, benign paroxysmal positional vertigo; BRV, benign recurrent vertigo; h, hour; MA, migraine with aura; MD, Meniere's disease; min, minute; MoA, migraine without aura; NA, not available; VM, vestibular migraine; SD, standard deviation; VP, vestibular paroxysmia*.

### Right-to-Left Shunt in Patients From Different Groups

**Figure 2:**
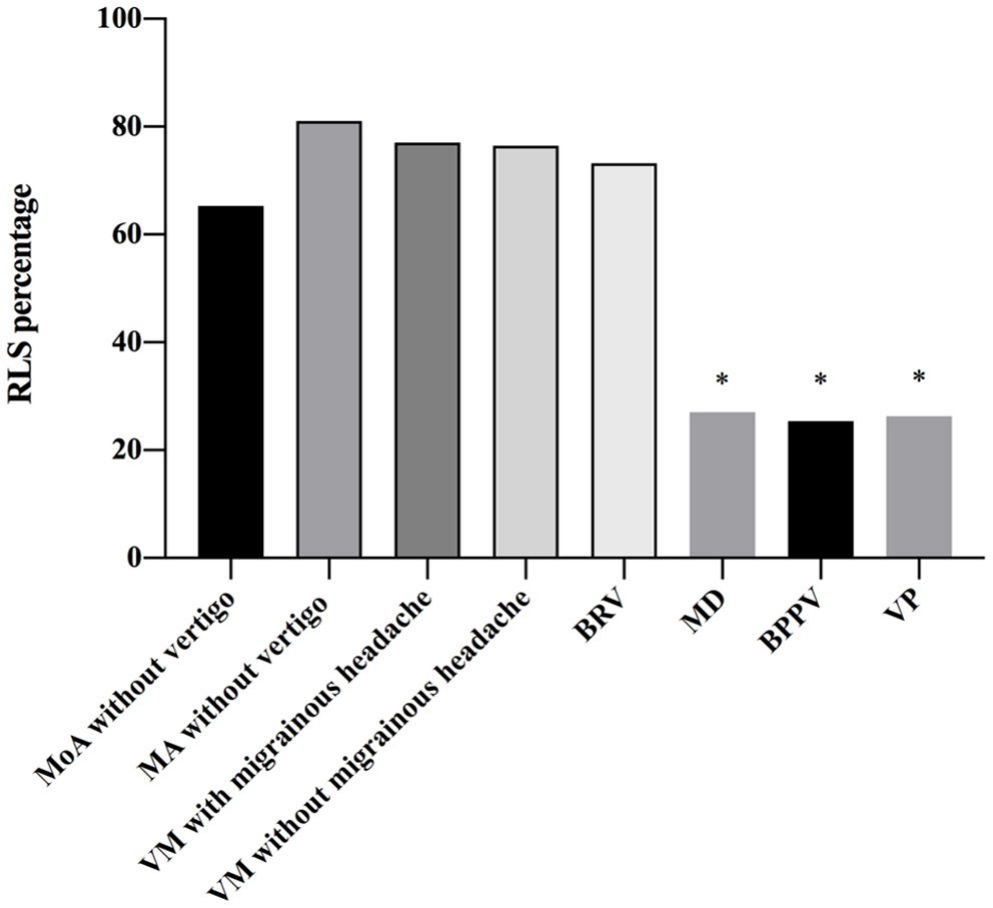
Frequency of right-to-left shunt (RLS) in patients with different diseases. There is a statistical difference between the frequency of RLS in patients with benign recurrent vertigo (BRV) and that in patients with Meniere's disease (MD), benign paroxysmal positional vertigo (BPPV), and vestibular paroxysmia (VP). The proportion of RLS in patients with BRV is significantly higher than that in patients with MD, BPPV, and VP (*P* < 0.05). The proportion of RLS in patients vestibular migraine (VM) with and without migrainous headaches is also significantly higher than that in patients with MD, BPPV, and VP (*P* < 0.05). However, there is no statistical difference between the frequency of RLS in patients with BRV and that in migraine without aura (*P* = 0.931), MA (*P* = 0.997), VM with migrainous headache (*P* = 0.787), and VM without migrainous headache (*P* = 0.754). BPPV, benign paroxysmal positional vertigo; BRV, benign recurrent vertigo; MA, migraine with aura; MD, Meniere's disease; MoA, migraine without aura; RLS, right-to-left shunt; VM, vestibular migraine; VP, vestibular paroxysmia. *P < 0.05.

The patients (61.98%) who were RLS-positive patients were detected by cTTE after the Valsalva maneuver. The proportion of RLS in patients with BRV and VM with and without migrainous headache was significantly higher than that in patients with Meniere's disease, BPPV, or VP (*P* < 0.05). There was no statistical difference between the frequency of RLS in patients with BRV and those with migraine without aura (*P* = 0.931), migraine with aura (*P* = 0.997), VM with migrainous headache (*P* = 0.787), and VM without migrainous headache (*P* = 0.754) (Table 3 and Figure 2).

**Table 3:** Right-to-left shunt and Dizziness Handicap Inventory in patients from different groups.

	**MoA without vertigo** **(*n* = 1,399)**	**MA without vertigo** **(*n* = 216)**	**VM with** **migrainous headache** **(*n* = 410)**	**VM without** **migrainous headache** **(*n* = 119)**	**MD** **(*n* = 148)**	**BPPV** **(*n* = 331)**	**VP** **(*n* = 57)**	**BRV** **(*n* = 71)**
RLS (%)	65.33	81.01	77.07	76.47	27.03	25.38	26.31	73.24
PFO	828	153	292	79	35	73	14	46
PAVM	57	12	19	9	2	8	1	4
PFO & PAVM	29	10	5	3	3	3	0	2
Grading of RLS								
0	485	41	94	28	108	247	42	19
I	263	33	68	17	17	40	7	12
II	257	54	106	34	14	30	3	18
III	394	88	142	40	9	14	5	22
DHI	NA	NA	46.33 ± 10.61	47.46 ± 9.40	51.26 ± 9.48	47.37 ± 13.42	50.69 ± 6.87	46.87 ± 10.37

*BPPV, benign paroxysmal positional vertigo; BRV, benign recurrent vertigo; DHI, Dizziness Handicap Inventory; MA, migraine with aura; MD, Meniere's disease; MoA, migraine without aura; NA, not available; PAVM, pulmonary arteriovenous malformation; PFO, patent foramen ovale; RLS, right-to-left shunt; VM, vestibular migraine; VP, vestibular paroxysmia*.

### Right-to-Left Shunt in Patients With Benign Recurrent Vertigo

The proportion of RLS in seven patients with BRV lasting <1 min was similar to those lasting for more than 1 min (71.43 vs. 73.44%). This was significantly higher than in VP (26.31%) and BPPV (25.38%). The proportion of RLS in patients with BRV aged <50 years and those aged ≥50 years was similar (73.08 vs. 73.68%), and the difference was not statistically significant.

### Association Between Right-to-Left Shunt Grade and Dizziness Handicap Inventory Scores

**Figure 3:**
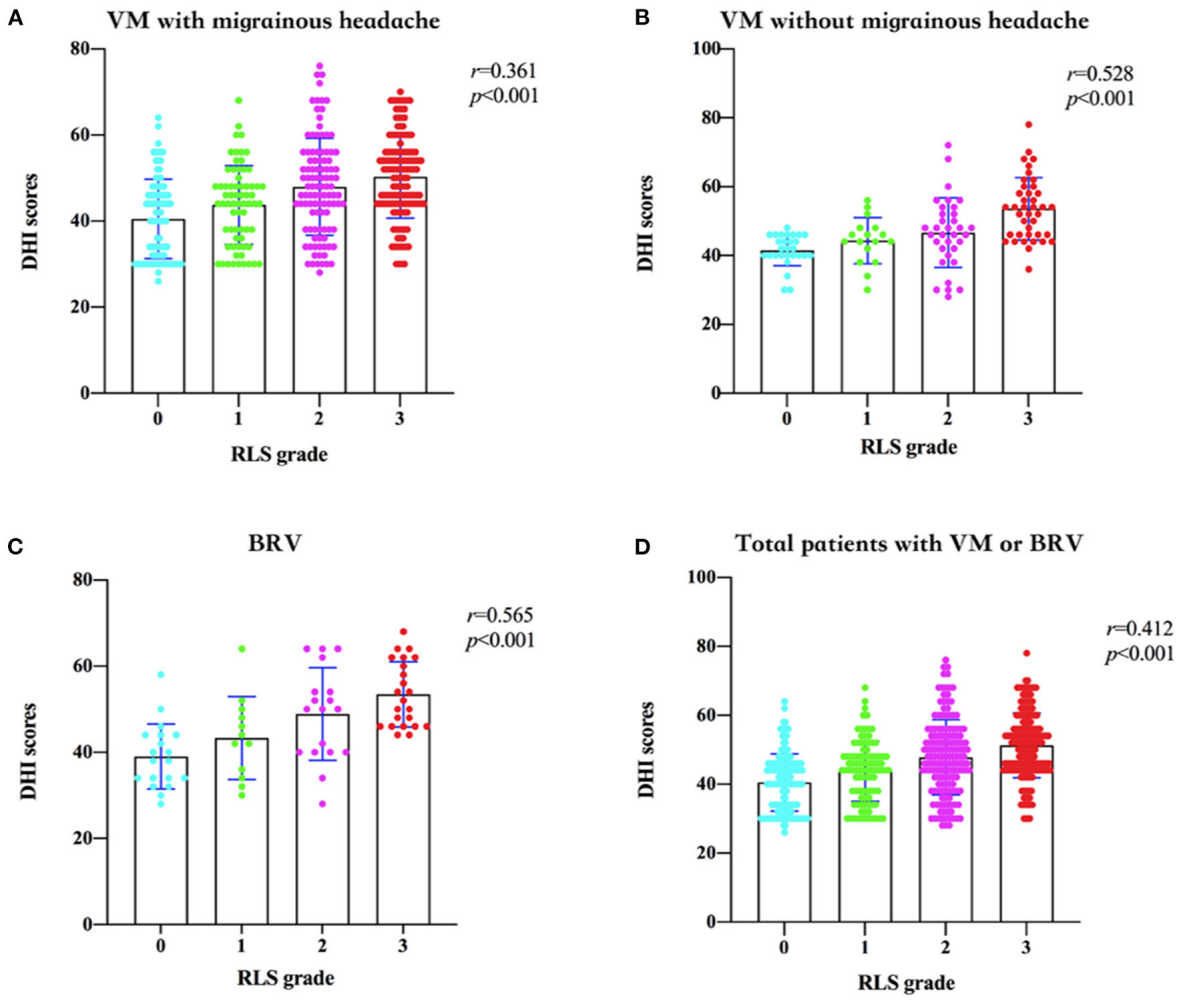
Dizziness Handicap Inventory (DHI) scores and the right-to-left shunt (RLS) grades in patients with vestibular migraine (VM) and benign recurrent vertigo (BRV). **(A)** In patients with VM with migrainous headaches, the DHI scores increase with increasing RLS grades, and there is a positive correlation between them. **(B)** In patients with VM without migrainous headaches, the DHI scores increase with increasing RLS grades, and there is a positive correlation between them. **(C)** In patients with BRV, the DHI scores increase with increasing RLS grades, and there is a positive correlation between them. **(D)** In all patients with VM or BRV, the DHI scores increase with increasing RLS grades, and there is a positive correlation between them. DHI, Dizziness Handicap Inventory; RLS, right-to-left shunt; VM, vestibular migraine; BRV, benign recurrent vertigo.

Based on the semi-quantitative classification of RLS, the DHI scores of grades 0, I, II, and III of patients from different groups are shown in Table 3. The RLS grade and DHI scores in patients with VM with migrainous headache (*r* = 0.361, 95% confidence interval [CI] 0.27–0.44), without migrainous headache (*r* = 0.528, 95% CI 0.38–0.65), and BRV (*r* = 0.565, 95% CI 0.38–0.71) are shown in Figures 3A–C, respectively. The DHI scores and RLS grades of all patients with VM or BRV (*r* = 0.412, 95% CI 0.34–0.48) are presented in Figure 3D. In patients with VM and BRV, the DHI scores increased with increasing RLS grades, and there was a positive correlation between them (*P* < 0.01).

## Discussion

Common paroxysmal vertigo can be categorized based on the following etiologies: central and peripheral. The most common central causes are VM and transient ischemic attack, and the most common peripheral causes are BPPV, Meniere's disease, and VP. VM is a separate disease entity with a genetic predisposition to recurrent dizziness or vertigo, with or without headache (23, 24). The BRV selected in previous clinical studies did not rule out a history of migraine and migraine-related symptoms; therefore, a considerable number of BRV cases may have met the diagnostic criteria for VM (6, 7). To avoid confusion, the selected BRV cases in this study only showed paroxysmal vertigo, that is, pure BRV. The selected patients with VM without headache could be differentiated from pure BRV by its migraine characteristics, such as photophobia, phonophobia, or visual aura. This study's results demonstrated, for the first time, that patients with BRV had a significantly higher proportion of RLS than those with peripheral vertigo diseases such as Meniere's disease, BPPV, or VP. Moreover, there was no statistical difference between RLS frequency in patients with BRV and those with VM and migraine. It is unknown whether BRV is a peripheral or central disorder. There have been some efforts to further categorize patients with BRV according to their etiology. For instance, Lee et al. described a new type of BRV with headshaking nystagmus of central origin (15). Although there were no central symptoms of photophobia, phonophobia, or visual aura, we speculate whether the central mechanisms are involved in pure BRV, and this is worthy of further study to clarify this disease entity.

There is a dose-effect relationship between PFO and migraine. Larger PFO, permanent PFO, and anatomical variation of PFO can aggravate RLS, which is associated with migraine attacks (11, 24). Moreover, PFO is more associated with migraine with aura than migraine without aura. The correlation between RLS and migraine does not imply causality, and the mechanism between the two is not clear. The possible mechanism is that vasoactive substances and microemboli from the venous system directly enter systemic circulation without passing through the pulmonary circulation. Thereby causing transient hypoperfusion in the area supplied by the cerebral arteries or cortical spreading depression, activating the trigeminal neurovascular system, and causing migraine attacks (25, 26). The initiating mechanism of VM is considered to be similar to that of migraine because RLS not only induces migraine but also causes vertigo by inducing plasma extravasation in the inner ear (27). In addition, microemboli or vasoactive substances may induce cortical spreading depression and activate the caudal parabrachial nucleus, which receives both trigeminal receptors and vestibular nerve afferents, resulting in simultaneous vestibular and migraine symptoms (1, 28). Therefore, we speculate that RLS may be involved in the pathogenesis of VM.

Many studies have found that BRV is highly correlated with migraine or VM and may have similar pathogenesis (6, 29, 30). However, some research has suggested otherwise (31). The question of whether BRV is a separate entity remains controversial, but a considerable number of BRVs are free from inner ear dysfunction and migraine and, therefore, do not develop into Meniere's disease and VM, suggesting that there may be different mechanisms in this aspect of BRV (7).

This study showed that the proportion of RLS in patients with BRV, VM, and migraine was significantly higher than in those with peripheral vertigo disease, suggesting that RLS might play an important role in the pathogenesis of BRV, VM, and migraine. The current literature remains discordant as to whether a link exists between RLS grade and the degree of vertigo. We found that the DHI score increased with RLS, suggesting that RLS plays an important role in the degree of vertigo and quality of life of vestibular disorders. The proportions of RLS in patients with BRV and VM were similar to those of migraine, suggesting that the above-mentioned mechanisms may share some features in the pathogenesis of migraine.

When the related mechanism of migraine involves only the vestibular system, it can manifest as pure BRV, whereas when the vestibular system and trigeminal neurovascular system are simultaneously involved, it can manifest as VM. When only the trigeminal neurovascular system is involved, the symptoms may include migraine, suggesting that BRV and VM may be subtypes or equivalents of migraine. VM and pure BRV can thus be unified in the migraine category. In this study, the RLS of patients with BPPV and VP was significantly lower than that of patients with migraine, suggesting that RLS was not involved in the pathogenesis of these peripheral vertigo diseases. RLS can play a role in the differential reference index of paroxysmal vertigo. In the future, for patients with refractory VM and BRV, the evaluation, intervention, and treatment of RLS would be worthy of further exploration.

Previous publications in the literature have predominantly defined the duration of BRV as more than 1 min or, in some studies, even more than 5 min; this extended duration primarily distinguishes BRV from BPPV and VP, which each have a duration of <1 min. There are few reports of a BRV of <1 min. A previous study found six cases of BRV with migraine that were <1 min, and only one case of BRV without migraine was <1 min (6). In clinical practice, there are truly spontaneous recurrent vertigo episodes lasting <1 min. These patients do not meet the diagnostic criteria for BPPV, VP, and VM. The duration of BRV was not specifically limited in this study. The proportion of RLS between the patients with BRV lasting less and more than 1 min was similar and significantly higher than that of VP and BPPV. Thus, strictly defining the duration as a criterion for diagnosing BRV may be debatable, and the evaluation of RLS may help provide a reference for BRV diagnosis.

Owing to the low probability of migraine onset after age 50 (32), patients with headache-free BRV after the age of 50 years have a low probability of developing VM, while patients with BRV before age 50 years have the possibility of progressing to VM. Therefore, we regarded age 50 as the threshold for the intragroup (subgroup) analysis of BRV. This study's results showed that the proportion of RLS in patients with BRV before and after age 50 was not significantly different and was similar to that of migraine. This may be related to the fact that BRV rarely evolves into VM (33).

With the insight into VM and the gradual clarification of its concept, there is an overlap between the classical concepts of BRV and VM, leading to ambiguity in the concept of BRV; thus, the effective use of the concept of BRV in clinics is difficult. In this study, pure BRV was proposed for the first time, and it can be effectively distinguished from VM in the absence of migraine-related characteristics. Pure BRV can also be effectively distinguished from peripheral vertigo, such as Meniere's disease. Therefore, the concept of pure BRV is helpful for clinicians to further study the mechanism, clinical characteristics, and prognosis of the disease. The concept and classification of VM and BRV in the future are worthy of further discussion.

There are some limitations to this study. First, vertebral basilar transient ischemic attack, persistent postural-perceptual dizziness, epileptic vertigo, and paroxysmal ataxia were excluded from the selection criteria of BRV in this study, therefore, these paroxysmal vertigo diseases were not compared with RLS. Second, there was a selection bias because the final selected patients were from both headache clinic and vertigo clinic. Additionally, BRV with headshaking nystagmus has been reported to originate from the central nervous system, and in this study, BRV was not subdivided according to whether it was associated with headshaking nystagmus.

## Conclusions

This is a large multicentre study, and the findings show that RLS differs in different types of paroxysmal vertigo. The prevalence of RLS in pure BRV and VM was similar but significantly higher than that in Meniere's disease, BPPV, and VP. There was a positive correlation between the RLS grading and DHI among patients with BRV and VM. The existence of RLS is valuable in the etiological diagnosis of paroxysmal vertigo and may be used as a differential reference index for the paroxysmal vertigo.

## Data Availability Statement

The raw data supporting the conclusions of this article will be made available by the authors, without undue reservation.

## Ethics Statement

The studies involving human participants were reviewed and approved by the Ethics Committee of the Second Affiliated Hospital, School of Medicine, Zhejiang University. The patients/participants provided their written informed consent to participate in this study.

## Author Contributions

KL conducted the design and conceptualization of the study, interpretation of the data, and revising the manuscript. XZ wrote and designed the manuscript. XT analyzed and interpreted the data. WH, YX, JC, HJ, XX, TZ, LZ, and MP collected and organized the data. All authors contributed to the article and approved the submitted version.

## Funding

This study was funded by the Zhejiang Provincial Natural Science Foundation of China [Grant Nos. LY19H090025 and LQ15H090003] and the National Natural Science Foundation of China [Grant Nos. 82171561 and 82174132]. The funders had no role in the design of this study or in the interpretation or presentation of its results.

## Conflict of Interest

The authors declare that the research was conducted in the absence of any commercial or financial relationships that could be construed as a potential conflict of interest.

## Publisher's Note

All claims expressed in this article are solely those of the authors and do not necessarily represent those of their affiliated organizations, or those of the publisher, the editors and the reviewers. Any product that may be evaluated in this article, or claim that may be made by its manufacturer, is not guaranteed or endorsed by the publisher.

## Abbreviations

BPPV: benign paroxysmal positional vertigo
BRV: benign recurrent vertigo
cTTE, contrast transthoracic echocardiography DHI: Dizziness Handicap Inventory
MA: migraine with aura
MD: Meniere's disease
MoA: migraine without aura
PAVM: pulmonary arteriovenous malformation
PFO: patent foramen ovale
RLS: right-to-left shunt
VM: vestibular migraine
VP: vestibular paroxysmia.
